# Computational Analysis of Structure-Based Interactions for Novel H_1_-Antihistamines

**DOI:** 10.3390/ijms17010129

**Published:** 2016-01-19

**Authors:** Yinfeng Yang, Yan Li, Yanqiu Pan, Jinghui Wang, Feng Lin, Chao Wang, Shuwei Zhang, Ling Yang

**Affiliations:** 1 Key Laboratory of Industrial Ecology and Environmental Engineering (MOE), Department of Materials Sciences and Chemical Engineering, Dalian University of Technology, Dalian 116024, China; yinfengyang@yeah.net (Y.Y.); yqpan@dlut.edu.cn (Y.P.); jhwang_dlut@163.com (J.W.); fenglin_dut@yeah.net (F.L.); chaowang_dlou@163.com (C.W.); zswei@dlut.edu.cn (S.Z.); 2 Laboratory of Pharmaceutical Resource Discovery, Dalian Institute of Chemical Physics, Graduate School of the Chinese Academy of Sciences, Dalian 116023, China; yling@dicp.ac.cn

**Keywords:** 3D-QSAR, H_1_-antihistamines, docking, molecular dynamics

## Abstract

As a chronic disorder, insomnia affects approximately 10% of the population at some time during their lives, and its treatment is often challenging. Since the antagonists of the H_1_ receptor, a protein prevalent in human central nervous system, have been proven as effective therapeutic agents for treating insomnia, the H_1_ receptor is quite possibly a promising target for developing potent anti-insomnia drugs. For the purpose of understanding the structural actors affecting the antagonism potency, presently a theoretical research of molecular interactions between 129 molecules and the H_1_ receptor is performed through three-dimensional quantitative structure-activity relationship (3D-QSAR) techniques. The ligand-based comparative molecular similarity indices analysis (CoMSIA) model (*Q*^2^ = 0.525, *R*^2^_ncv_ = 0.891, *R*^2^_pred_ = 0.807) has good quality for predicting the bioactivities of new chemicals. The cross-validated result suggests that the developed models have excellent internal and external predictability and consistency. The obtained contour maps were appraised for affinity trends for the investigated compounds, which provides significantly useful information in the rational drug design of novel anti-insomnia agents. Molecular docking was also performed to investigate the mode of interaction between the ligand and the active site of the receptor. Furthermore, as a supplementary tool to study the docking conformation of the antagonists in the H_1_ receptor binding pocket, molecular dynamics simulation was also applied, providing insights into the changes in the structure. All of the models and the derived information would, we hope, be of help for developing novel potent histamine H_1_ receptor antagonists, as well as exploring the H_1_-antihistamines interaction mechanism.

## 1. Introduction

Insomnia, defined as the subjective perception of difficulty with sleep initiation, is a prevalent health complaint [1]. The characteristics of insomnia are difficulty falling or maintaining asleep at night and increased fatigue in the daytime. Multiple researchers have found that the risk to develop insomnia increases with aging [2]. An astounding 20%–33% of the adult population is affected by insomnia symptoms, with an estimated 10% to 15% meeting the criteria of diagnosing insomnia disorder [3]. In addition, insomnia in later life can result in negative consequences, including decreased quality of life, impairment in function in general, increased risk for falls and nursing home placement [4]. However, many things about this highly prevalent insomnia, including its function mechanism, clinical course, causes, *etc.*, still remain unknown [5]. Consequently, pharmacologic agents are becoming increasingly desirable for a convenient option for the treatment of insomnia.

Histamine predominates in a series of physiological processes, like in the functions of immune cells, gastrointestinal tract and CNS (central nervous system). For instance, histamine in CNS is closely connected with a wide spectrum of functions, like the regulation of arousal and the sleep-wake cycle, appetite control, satiation, nociception and cognition [6]. Antihistamines were first introduced in the 1940s and represent one of the most commonly-used medications today [7]. This is just due to the specific histamine-blocking capability and marked sedative effects; H_1_-antihistamines are often used for sleep promotion nowadays [8]. Meanwhile, in quite a few medications, anti-histamines are also applied for the treatment of allergies, cold symptoms, nausea, itching and insomnia. However, after bedtime use of anti-histamines, the long plasma half-lives and protracted CNS exposure always are assumed to cause the frequently-occurring next-day impairment, which in turn suggests the importance of an improved pharmacokinetic profile in the selection of new insomnia-aimed H_1_-antihistamines [9]. As a result, for the treatment of insomnia, the use of novel and selective H_1_-antihistamines with appropriate exposure is promising as an alternative to current medications, especially for sleeping improvements during the latter third of the night and overall sleep efficiency [10]. Such agents increase sleep time and improve sleep efficiency [10].

By combining with and stabilizing the inactive conformation of H_1_-receptors, H_1_-antihistamines act as inverse agonists and, thus, in this way, interfere with the actions of histamine at H_1_-receptors [11]. As a matter of fact, the development of H_1_-antihistamines has undergone three generations. The first-generation antihistamines, which are also known as sedative antihistamines, contain brompheniramine, chlorphenamine and mepyramine (an ethylenediamine), hydroxyzine (a piperazine), diphenhydramine (an ethanolamine), promethazine (a phenothiazine) and triprolidine (alkylamine derivative) [12]. Despite the wide application of these medications for insomnia therapy, publications about placebo-controlled trials concerning the agents’ safety and efficacy properties are still hard to find [12]. However, the side effects are obvious for the first-generation H_1_-antihistamines: (1) because of their poor receptor selectivity and high penetration rate of the blood-brain barrier, a depression of the CNS appears, which always leads to many symptoms, like not only drowsiness, fatigue, somnolence and dizziness, but also impairments of cognitive function, memory and psychomotor performance; (2) cardiac problems also appear due to anti-muscarinic effects, blockade of cardiac ion currents and α-adrenergic-receptor blockade; and (3) other problems occur owing to the ability to block α-adrenergic, muscarinic and serotoninergic receptors, which in turn bring about dry eyes, dry mouth, mydriasis, urinary retention, constipation, erectile dysfunction, gastrointestinal motility and memory deficits [13]. On the contrary, hundreds of clinical trials lasting weeks/months (with proper randomized, double-masked, placebo-controlled properties) supported the second-generation H_1_-antihistamines for the treatment of seasonal/intermittent and perennial/persistent allergic rhinoconjunctivitis and chronic urticaria. In these trials, both the inclusion and exclusion criteria were clearly stated, with an adequate number of participants enrolled and attrition and adherence appropriately documented [14]. As for the third-generation, this was usually applied to describe some new antihistamines that are selective isomers or active metabolites of the older second-generation antihistamines. The similarity of the second and third generations is that they are both devoid of the first generation’s side effects, while exhibiting remarkable anti-histamine activity at the same time [15]. Nevertheless, some second- and third-generation antihistamines can result in serious and often fatal cardiac arrhythmias, although they are potent inhibitors of the human ether-a-go-go (hERG) channel, which is associated with the prolongation of cardiac QTc [16,17]. Accordingly, for treating insomnia, an alternative to current medications, like selective H_1_-antihistamines with an appropriate duration of CNS exposure, is a matter of choice, and investigating novel antihistamine antagonists is therefore very important.

In the process of finding rational small-molecule inhibitors, computational (rational) drug design has made significant contributions to the discovery of new and more efficacious therapeutics [18]. Computational drug discovery and optimization approaches, based on quantitative structure-activity relationship (QSAR) principles, offer an efficient, as well as an economical alternative for drug development when employed in conjunction with synthetic medicinal chemistry and experimental testing of lead compounds [19,20,21,22]. Series of 3D-QSAR methods, including especially comparative molecular field analysis (CoMFA) and comparative molecular similarity indices analysis (CoMSIA), have proven their efficiency in aiding drug design over the last few years [23,24,25]. In this work, in order to investigate the interactions of 129 novel H_1_-antihistamines synthesized by Graham *et al.* [26,27,28,29,30,31,32,33], we jointly used 3D-QSAR, docking and molecular dynamics (MD) simulation approaches, to perform a deep exploration for those physical-chemical factors impacting the H_1_-antihistamines’ bioactivity and a detailed investigation for understanding the binding mode of the histamine H_1_ receptor. The established results are expected to provide valuable insights into potential structural modifications for developing more potent and selective H_1_-antihistamines, as well as understanding their interaction mechanisms.

## 2. Results

### 2.1. 3D-QSAR Analysis

**Figure 1:**
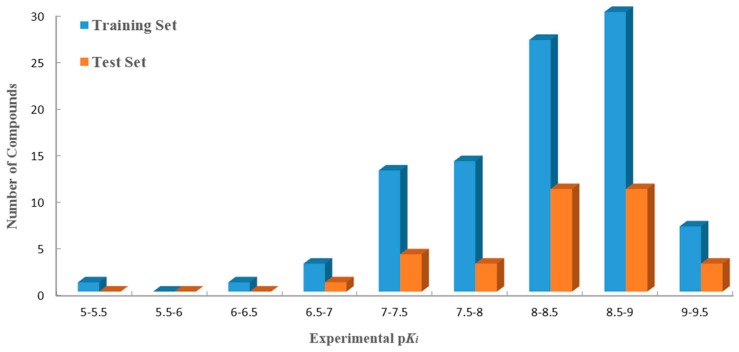
Distribution of activities (p*K_i_*) for the training and the test sets *versus* the numbers of compounds. The training and the test sets are colored blue and orange, respectively.

The predictive capability of the models relies on the alignment rules. Thus, various alignment strategies can lead to different statistical values in the constructed QSAR models [34]. Among the three alignment methods (Alignment-I, -II and -III) described in our study, the atom-based alignment (Alignment-I) has the best predictive ability (with the largest *Q*^2^, *R*^2^_ncv_, *R*^2^_pred_ and the lowest standard error of estimate (SEE), standard error of prediction (SEP) values). Therefore, the Alignment-I method was finally chosen for the 3D-QSAR analysis. In addition, the PLS method with LOO analysis can illustrate the reliability of a 3D-QSAR model and, thus, was used for the prediction and design of new molecules. Furthermore, several statistical parameters, including *Q*^2^, *R*^2^_ncv_, *R*^2^_pred_, SEE, SEP, F-statistic and the optimal number of principal components (*N*), were also analyzed. In order to evaluate the *R*^2^_pred_ value, the whole dataset was divided into training (96 compounds) and test sets (33 ones). The distribution of the activity data of all 129 compounds with their numbers is shown in Figure 1.

By the analysis of the obtained QSAR models, the CoMFA model combined with steric and electrostatic fields was obtained. As we know, *Q*^2^ (>0.5) is one of the best parameters for evaluating whether a good 3D-QSAR model is acceptable. However, as depicted in Table 1, two descriptor fields in CoMFA form all three possible combination models, *i.e.*, steric (S), electrostatic (E) and SE models. The *Q*^2^ values of these models are 0.013, −0.081 and 0.044, respectively, which do not meet the criterion (*Q*^2^ > 0.5), indicating an unacceptable 3D-QSAR model. Thus, the CoMSIA models with a combination of five descriptor fields, including S, E, hydrophobic (H), H-bond donor (HB-donor) and acceptor (HB-acceptor), were developed to generate the optimal 3D-QSAR model.

**Table 1:** Summary of comparative molecular field analysis (CoMFA) and comparative molecular similarity indices analysis (CoMSIA) results. S, steric; E, electrostatic; H, hydrophobic.

PLS Statistics	*N*	*Q*^2^	SEP	*R*^2^_ncv_	SEE	*F*	*R*^2^_pred_	Contribution (%)				
								S	E	H	D	A
***CoMFA***												
*S*	1	0.013	0.730	0.214	0.652	25.618	0.020	100	–	–	–	–
*E*	1	−0.081	0.764	0.088	0.702	9.052	0.204	–	100	–	–	–
*SE*	4	0.044	0.730	0.563	0.494	29.325	0.064	50.2	49.8	–	–	–
***CoMSIA***												
*S*	1	−0.223	0.628	0.213	0.504	8.365	0.272	100	–	–	–	–
*E*	4	0.224	0.659	0.601	0.472	34.296	0.450	–	100	–	–	–
*H*	5	0.132	0.700	0.581	0.487	24.921	0.177	–	–	100	–	–
*D*	4	0.126	0.698	0.164	0.683	4.450	0.075	–	–	–	100	–
*A*	3	−0.011	0.747	0.271	0.634	11.389	0.005	–	–	–	–	100
*SE*	5	0.196	0.680	0.661	0.437	35.113	0.503	20.5	79.5	–	–	–
*SH*	7	0.116	0.714	0.734	0.392	34.635	–	27.5	–	72.5	–	–
*SD*	4	0.212	0.663	0.366	0.595	13.116	−0.07	36.8	–	–	63.2	–
*SA*	3	−0.01	0.743	0.329	0.608	15.057	−0.022	27.4	–	–	–	72.6
*EH*	4	0.297	0.501	0.913	0.177	73.179	0.920	–	66.7	33.3	–	–
*ED*	2	0.197	0.518	0.583	0.373	21.010	–	–	75.2	–	24.8	–
*EA*	5	0.255	0.525	0.906	0.187	51.889	0.913	–	56.8	–	–	43.2
*HD*	6	0.044	0.606	0.771	0.297	14.615	0.789	–	–	89.6	10.4	–
*HA*	3	0.226	0.517	0.802	0.261	39.174	–	–	–	33.6	–	66.4
*DA*	2	0.186	0.521	0.642	0.346	26.847	0.670	–	–	–	27.5	72.5
*SEH*	5	0.260	0.524	0.936	0.154	78.990	0.940	10.8	60.0	29.2	–	–
*SED*	2	0.143	0.535	0.597	0.366	22.240	0.628	11.2	65.6	–	23.2	–
*SEA*	5	0.261	0.523	0.908	0.185	53.165	0.915	11.3	50.5	–	–	38.2
*SHD*	6	−0.115	0.655	0.819	0.264	19.552	0.832	28.2	–	59.9	11.9	–
*SHA*	2	0.169	0.526	0.675	0.329	31.120	0.699	11.1	–	23.8	–	65.1
*SDA*	2	0.149	0.533	0.655	0.339	28.530	0.682	10.5	–	–	25.1	64.4
*EHD*	6	0.223	0.547	0.939	0.154	66.188	0.943	–	60.0	30.5	9.4	–
*EHA*	4	0.284	0.506	0.905	0.184	66.697	0.913	–	43.3	21.8	–	35.0
*EDA*	3	0.206	0.523	0.831	0.242	47.381	0.844	–	41.3	–	14.3	44.3
*HDA*	4	0.262	0.642	0.557	0.497	28.630	0.268	–	–	31.2	37.9	30.9
***SEHD***	**9**	**0.525**	**0.529**	**0.891**	**0.253**	**78.468**	**0.807**	**9.6**	**43.7**	**24.5**	**22.2**	**–**
*SEHA*	6	0.287	0.642	0.788	0.348	55.196	0.483	9.0	42.7	23.5	–	24.8
*SEDA*	9	0.324	0.632	0.854	0.293	55.962	0.600	10.4	44.7	–	20.5	24.4
*SHDA*	4	0.238	0.652	0.554	0.499	28.202	0.241	9.7	–	25.8	37.3	27.3
*EHDA*	9	0.433	0.579	0.893	0.251	79.732	0.709	–	36.5	21.3	19.8	22.4
*SEHDA*	9	0.445	0.572	0.898	0.246	83.748	0.720	6.7	34.8	18.6	19.3	20.6

*Q*^2^, cross-validated correlation coefficient after the leave-one-out procedure; *R*^2^_ncv_, non-cross-validated correlation coefficient; SEE, standard error of estimate; *F*, ratio of *R*^2^_ncv_ explained to unexplained = *R*^2^_ncv_/(1 − *R*^2^_ncv_); *R*^2^_pred_, predicted correlation coefficient for the test set of compounds; SEP, standard error of prediction; *N*, optimal number of principal components; S, Steric; E, electrostatic; H, hydrophobic; D, HB-donor; A, HB-acceptor.

Five descriptor fields (S, E, H, HB-donor and-acceptor) with all 31 possible combinations were calculated to build the optimal 3D-QSAR model in the CoMSIA analysis. The obtained *Q*^2^, *R*^2^_ncv_ and *R*^2^_pred_ values and other statistical results from the CoMSIA analysis for all combinations of the fields are depicted in Table 1. From Table 1, we can see that the CoMSIA model based on S, E, H and HB-donor fields was the best model, yielding the best *Q*^2^, *R*^2^_ncv_, SEE, *F* and *R*^2^_pred_ values, and, thus, was chosen for further study. This model gives good statistical parameters and has the optimal number of components (*N* = 9) with a *Q*^2^ of 0.525, which is better than the other value from the previous CoMFA model. In addition, the high *R*^2^_ncv_ and *F*-value of 0.891 and 78.468 along with the low SEE value of 0.253 indicate that the CoMSIA model with the S, E, H and HB-donor is statistically satisfactory. The contributions of the relative S, E, H and HB-donor fields were 9.6%, 43.7%, 24.5% and 22.2%, respectively. The results indicate that the E field plays an important role of impacting the activity of H_1_-antihistamines. The H and HB-donor fields contribute equally to the majority of the activity.

**Figure 2:**
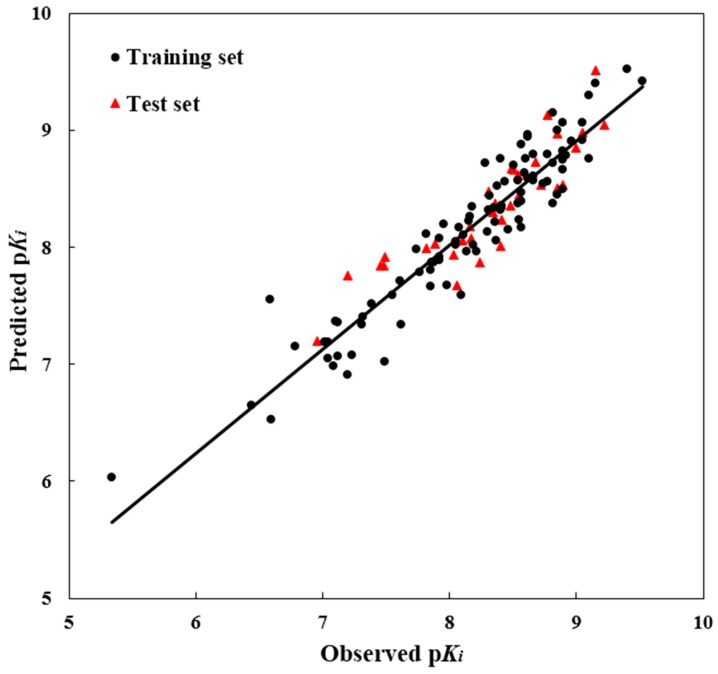
The ligand-based correlation plots of the predicted *versus* the actual p*K_i_* values using the training (filled red triangles) and the test (filled black dots) set compounds based on the optimal CoMSIA model.

To test the predictability of a 3D-QSAR model, a crucial step is to examine the quality of a model for the prediction of the biological activity of the compounds. Therefore, for the purpose of assuring the predictive power of the model built from the training set, the test set modeling was constructed to predict the bioactivities values of the 39 compounds in the test set. For the test molecules, their predicted and actual bioactivity values were in good accordance within a tolerable error range. Additionally, the *R*^2^_pred_ value was computed as 0.807, showing good external prediction performance of the obtained model. The good correlation between the predicted and observed activities p*K_i_* values for all molecules is shown in Figure 2, indicating the strong predictive ability of the obtained optimal CoMSIA models. To sum up, the comprehensive assessment gives satisfactory results, showing a high precision of prediction from the built 3D-QSAR model with actual biological validation values.

### 2.2. Graphical Interpretation of CoMSIA Model

**Figure 3:**
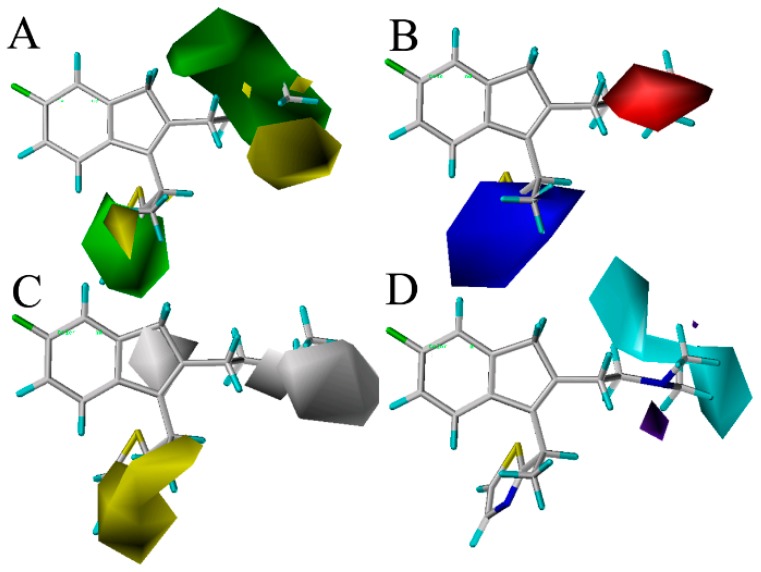
Contour maps of CoMSIA combined with compound 49. (**A**) Contour maps in steric (green/yellow) fields. Green and yellow contours represent regions where bulky groups will increase and decrease the activity, respectively; (**B**) Contour maps in electrostatic (red/blue) fields. Red and blue contours represent regions where negative- and positive-charged substituents will decrease and increase the activity, respectively; (**C**) Contour maps in hydrophobic (yellow/gray) fields. Yellow and gray contours represent regions where the hydrophobic and hydrophilic groups will increase their activity; (**D**) Contour maps in H-bond (HB) donor (cyan/purple) fields. Cyan and purple contours represent regions where HB donor substituents will enhance and decrease the activity, respectively.

In order to reveal the crucial characteristics of the ligands, four contour maps were generated from the optimal ligand-based CoMSIA model. Generally speaking, the painted contours around the lattice points where the 3D-QSAR model strongly correlated changes in the ligands’ region values with changes in biological efficiency. This is especially important to increase or decrease the activity of the ligand by changing the molecular structure traits leading to the interaction between the binding sites of the receptor and the ligand [35]. To visualize the results of the CoMSIA model, the most potent compound 49 in the whole dataset was overlapped on the CoMSIA contour maps. Figure 3 shows the CoMSIA contour maps of the four description fields, including S, E, H and HB-donor. The contribution of the positive and negative standards was kept at the default values of 20% and 80%, respectively.

Figure 3A displays the CoMSIA steric contour maps in which yellow and green contours indicate that the bulky groups are disfavored and favored regions for the activity, respectively. From the figure, we can see that the position-16 of the molecule 49 is surrounded by a green isopleth, indicating that the compounds with a big substituent at this position would be more active than the other compounds with a smaller or without a group at the same position. The fact that the compound 79 (p*K_i_* = 8.620) with –(CH_2_)_2_Ph is more active than the compound 77 with –Ph (p*K_i_* = 7.094) at this position is a good instance. The higher activity of compound 96 with the thiazole substituent (p*K_i_* = 8.886) when compared to compound 87 with –CH_3_ (p*K_i_* = 8.620) is also such a case. As a result, in order to enhance the antagonism effect of the ligand, new analogs with bulk substituents in these positions should be explored. In the second place, the position-18 is covered by a big yellow contour, revealing that the bulky group at this position has an unfavorable effect on the ligand’s histamine H_1_ receptor antagonism activity. Indeed, this is in good agreement with the reported experimental results that compound 16 (p*K_i_* = 6.592) has a –CH_3_ substituent at this position and has less antagonism activity than compound 17 (p*K_i_* = 7.495) with –H.

The CoMSIA electrostatic contours are shown in Figure 3B, where the red and blue regions indicate the electronegative and positively-charged favorable region, respectively. In the positive electrostatic field, a blue contour near position-11 of the thiazole substituent indicates that the electropositive moiety at this position might enhance the biological activity for new compounds. Take compounds 33, 40, 41, 87 and 99 for examples; the strong and electropositive –OCH_3_ substituent at the position-3 of the thiazole ring results in significantly increased biological activity. In addition, when compared to compound 65 (p*K_i_* = 8.092), which has a positively-charged favorable for the corresponding oxygen near the position-14 of thiazole, it is obvious that the compound 66 (p*K_i_* = 8.174) has a higher activity with an electropositive charge favorable for the corresponding sulfur. At the same time, red contours in the surroundings of the NCH_3_CH_3_ substituents near the position-17 denote that electronegative substituents are favorable in this zone for an increase of the antagonism of the histamine H_1_ receptor. Due to this phenomenon, which may have something to do with the atoms N and O (electronegative charged atoms) of compounds 5, 19, 29, 32 to 35, 116 to 117 and 119 at the position-17, it can explain why a small red contour is depicted here.

Figure 3C depicts the CoMSIA hydrophobic contour maps, where the yellow and gray regions are favorably hydrophobic and hydrophilic for the activity, respectively. A yellow contour is found near the thiazole ring in the position-14, showing that the hydrophobic substituents (like –OMe, –F, –Cl) at this position will increase the activity. Compounds 7, 8, 9, 10, 13 and 14 with higher potency are observed to follow this mode closely for the presence of a hydrophobic group –OMe, –F and –Cl at the position. Moreover, a big gray isopleth near the NCH_3_CH_3_ substituent of the position-17 and a small contour located at position-16 indicate that the hydrophilic substituents at these positions will increase the activity.

The CoMSIA HB-donor contour maps are represented in Figure 3D. In the HB-donor field, the cyan and purple isopleths indicate the regions where the HB-donor substituents would enhance and decrease the activity, respectively. As shown in Figure 3D, a larger cyan isopleth around the NCH_3_CH_3_ group in the position-18 reveal that HB-donor groups at the position may benefit from the activity. Compound 60 (p*K_i_* = 8.553) has a higher activity than compound 52 (p*K_i_* = 8.143), and compound 75 (p*K_i_* = 8.569) is more potent than compound 76 (p*K_i_* = 7.124), which validate these results. Additionally, a small-sized purple contour is observed near the position-16 of the benzene group, which reveals that the HB-acceptor substituents at these positions will be unfavorable for the activity. This is in agreement with the observations that 88 (p*K_i_* = 7.851) is less potent than 119 (p*K_i_* = 8.310), because 88 has nitrogen as an HB-acceptor substituent, which is far from the purple region, while 119 with nitrogen is lightly close to this area, favorable for potency.

Taken together, the results described above reveal the requisite structure for improving the bioactivities of the antagonists of this class.

### 2.3. Molecule Docking

Docking is a crucial method for probing the relationships between the ligand of the receptor target. In order to explore the interactions between a compound and a histamine H_1_ receptor, useful information about the structural features of binding sites of proteins can be provided by the molecular docking procedure, which is of help for developing new high potency antagonists. Currently, all of the ligands in the dataset were docked into the active site of the histamine H_1_ receptor for the purpose of investigating the action mechanism of the H_1_-antihistamines with their target.

#### 2.3.1. Docking Validation

**Figure 4:**
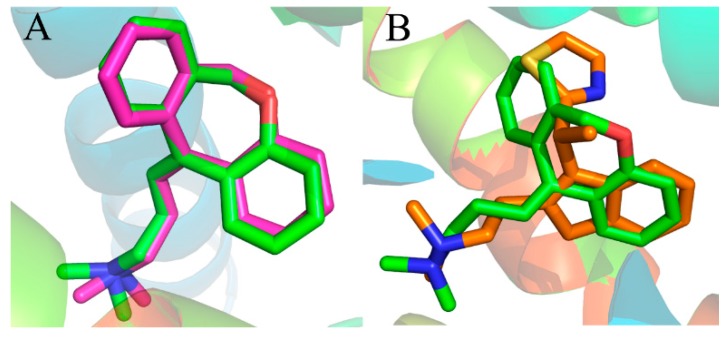
(**A**) Binding poses of co-crystallized (magenta) and re-docked (green) compound doxepin; (**B**) overlap of the compound 49 (orange) and experimental doxepin (green; PDB code: 3RZE) conformation.

Prior to the docking project, the reliability of the docking method is determined by the resemblance of docked conformation with the corresponding co-crystallized ligand. Presently, the X-ray crystal structure of the histamine H_1_ receptor complex was used to support the reliability of the docking study. The known co-crystallized doxepin [36] was removed from the complex of the crystalline structure and re-docked into the active site of the histamine H_1_ receptor. The most possible binding conformation is the docked pose with the highest total score value. Figure 4A shows the good superimposition of the original co-crystallized conformation with the re-docked doxepin, indicating that the reliability of the docking procedure is acceptable. Meanwhile, the simulated compound 49 was superimposed with experimental poses of doxepin in the binding site (Figure 4B). From the superimposition, only a slight difference can be observed between the two compounds, validating the fact that the docking procedure is a reliable method.

#### 2.3.2. Ligand-Binding Pocket

**Figure 5:**
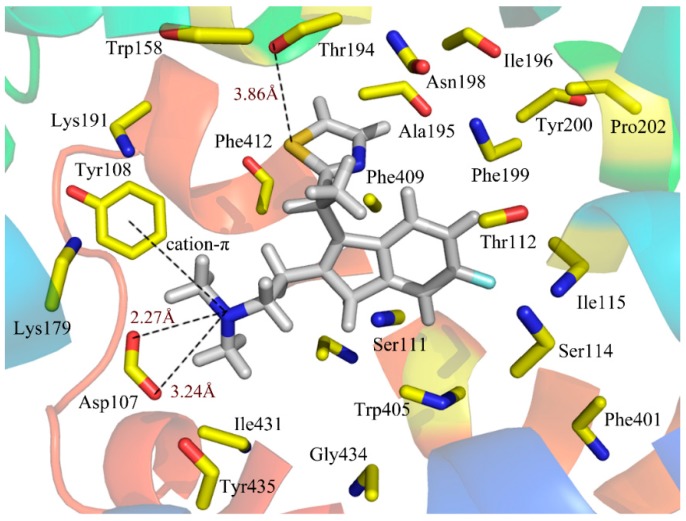
Docked conformation of compound 49 into histamine H_1_ receptor. The projection highlights the structure of the active site with compound 49, which is displayed in sticks.

To see how an antagonist can anchor at the histamine H_1_ receptor, we tried to dock antagonist into the binding cavity assigned by the compounds that affect histamine binding. We mainly concentrated on the relationships between the receptor and the ligand for the key structures of the most active compound 49. Figure 5 illustrates the binding mode of compound 49 docked into the histamine H_1_ receptor. The obtained binding conformation shows the main interactions with the receptor binding site. Obviously, it is observed that the lipophilic and hydrophobic interaction play important roles in the binding of the ligands to their target histamine H_1_ receptor. As shown in Figure 5, the thiazole ring can be seen among the lipophilic reciprocity scope of Trp158 and was away from Lys191 in the entrance of the active site [37]. A lipophilic region with the Phe412, Tyr435 and Trp158 is distributed in favorable stations and participates in the ligand-receptor binding [38]. In addition, the aromatic of compound 49 is surrounded by a hydrophobic region, including Phe409, Phe199, Phe401, Ala195, Tyr200, Pro202, Ile196 and Trp405, as well as a blend of hydrophilic amino acids Thr112 and Ser114. The mainly conserved residues were surrounded with compound 49 contain Ile115, Tyr435 and Gly434. The non-conserved residues Trp158 and Asn198 make little hydrophobic contribution to the ligand. All of these results match well with the docking results from the studies of Tatsuro *et al.* [37].

In addition to the interactions described above, H-bond interactions were also calculated in the docking study. Generally speaking, an H-acceptor distance less than 3.5 Å with a donor-H-acceptor angle larger than 120° is the geometric criterion for forming the H-bonds [39]. An H-bond with a distance in the scope of 3.2 and 4.0 Å is considered a weak H-bond [39]. According to Figure 5, Asp107, a strictly-conserved residue in the histamine H_1_-receptor, forms a salt bridge with the compound 49 (cationic nitrogen). It is also reported that this interaction plays an important role in forming the binding conformation [40,41,42]. Moreover, this cationic nitrogen is engaged in the π-stacking with Tyr108, which is consistent with the studies of Wang *et al.* [43]. In addition, a strong H-bond was formed between the S atom of the thiazole and the Thr194 (–S···HO, 3.86Å), which firmly stabilizes the ligand-receptor binding interaction and makes the thiazole ring perfectly clamped with the ligand in the binding conformation. As a result, the conclusion can be safely drawn that the ligands adjusted to the lipophilic, hydrophobic, salt bridge and H-bond interactions would act as potential antagonists.

### 2.4. Comparing the Results of 3D Contour Maps with Docking

Figure 5 shows that the NCH_3_CH_3_ group and thiazole ring near the position-17 of compound 49 are located at the bottom, and the entry of this pocket is formed by the target protein interface, respectively. Especially, the thiazole ring occupies the most opened area of the entry. As seen from Figure 3A, the bulky groups introduced into the field surrounding the NCH_3_CH_3_ group and the thiazole ring are favorable for improving the binding affinity with the protein, which coincide with this docking result. Moreover, the cavity seems deep enough to accommodate bulkier groups at the thiazole ring position, which would be conducive to the interactions with the protein. It is worth noting that electropositive residues Lys179 and Lys191 surrounding the position-15 suggest that electropositive substituents introduced into the antagonists are favorable for the ligand activity, the conclusion of which is in agreement with the CoMSIA electrostatic blue contour results (Figure 3B). As shown in Figure 3C, introduction of suitable electronegative groups into the NCH_3_CH_3_ group region would capture an additional interaction with Asp107, which is corroborated by the fact that this electronegative favorable area is surrounded by the red contour of the CoMSIA model. Therefore, the conclusion of the crucial interactions’ formation with the negatively-charged moieties of ligands is also inferred from the blue contour results by CoMSIA analysis.

The active site of the histamine H_1_-receptor also reaches good agreement with the above hydrophobic contour maps of the CoMSIA model. The thiazole ring is tightly located next to a relatively large hydrophobic groove consisting of the hydrophobic residues Phe412, Phe409, Phe199, Trp158, Ala195 and Ile196. Remarkably, in this position, the space is large and open enough to hold a large hydrophobic group in the surface-exposed hydrophobic pocket, which coincides with the large hydrophobic contour map depicted in (Figure 3C). Meanwhile, residues Gly434 and Ser111, hydrophilic in nature, around the NCH_3_CH_3_ moiety indicate that antagonists with hydrophilic substituents in this position may cause the reduction of the activity. These results are also explained by the previous hydrophilic contour maps of the CoMSIA model.

Consequently, according to the docking results, the steric, electrostatic and hydrophobic fields in the binding pocket of the histamine H_1_-receptor are without a doubt interrelated with the contour maps, revealing that the QSAR model is reliable and can provide better suggestions to help with potent H_1_ antagonists’ design.

### 2.5. MD Simulations

In a molecular docking simulation, the compound, the small molecule ligand, is considered to be the flexible body, while the receptor protein is taken as the rigid or partially-flexible one. Nevertheless, molecular dynamics analysis treats both the ligand and protein as elastic to provide an attractive alternative by structural refinement, in which the conformation is adjusted upon the ligand binding for the whole system. Generally, in an MD simulation, compared to using a single crystal structure, an average structure of the last 1 ns employed in the simulation is considered more reliable. In the present work, to further discover a dynamic plot of the conformation changes that happen in aqueous solution and both the ligand and receptor, a 5000-ps MD simulation was performed according to the docked complex, including the histamine H_1_-receptor and the most potent compound 49.

**Figure 6:**
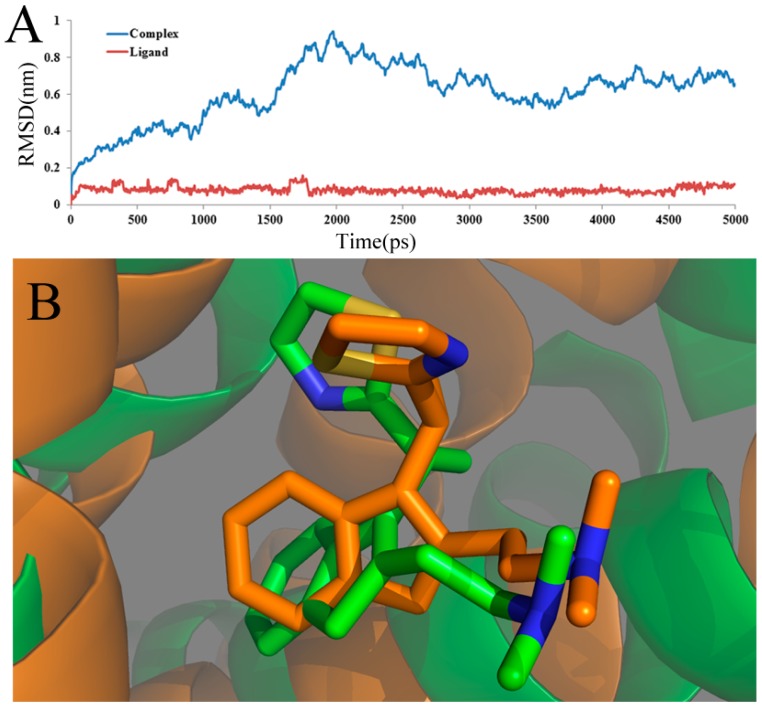
(**A**) Plot of the root-mean-square deviation (RMSD) of docked complex/ligand *versus* the MD simulation time in the MD-simulated structures; (**B**) view of the superimposed backbone atoms of the average structure for the MD simulations and the initial structure of the docking for the complex. Compound 49 is represented as a carbon-chain in green for the initial complex and a carbon-chain in orange for the average structure, respectively.

To ensure the dynamic stability and rationality of the chosen conformations, the root-mean-square deviation (RMSD) values of the atoms of the protein backbone were calculated. The structural drift from the initial coordinates, as well as the atomic fluctuation throughout the whole MD simulation process are measured directly by RMSD. The RMSD backbone atoms of the complex and ligand about the starting systems are summarized and shown in Figure 6A. In this figure, until 1.2 ns, the ligand-receptor complex (blue line) shows a backbone RMSD of ∼0.2 to ∼0.6 nm during this part of the simulations. After 1.2 ns, the complex structure exhibits minimum deviation until the end of simulation, that is 3 ns with its backbone RMSD ranging from ∼0.6 to∼0.9 nm, whereas the ligand-receptor complex shows maximum deviation. After 3 ns, it is shown that the three-dimensional structures of the protein have no significant change, and the RMSD of the complex, reaching about 0.32 nm, remains stable over the simulation process. This stability clearly suggests that the docked complex structure is in a metastable conformation starting from 3 ns of simulation. What is more, after 3 ns, the RMSD of the ligand, shown as a red line, reaches a stable value (about 0.13 Å), revealing that the structure of the ligand is in a stable conformation after 3 ns of simulation. Overall, low RMSD values over time for the complex and ligand imply the stability of the structure over time and, thus, the overall accuracy of the topology.

Furthermore, the superimposed result of the initial docked structure and the last 1 ns average MD simulated structure are shown in Figure 6B, where the initial docked complex structure is displayed as a green line, and the average structure of simulation is displayed as an orange line. Additionally, the green and orange sticks represent the original and the final average structures of the ligand, respectively. Clearly, it is found that that docked complex of the most active compound 49 shows more consistency in conformation as deduced from the low deviation in the RMSD after a certain time during the MD simulation study.

**Figure 7:**
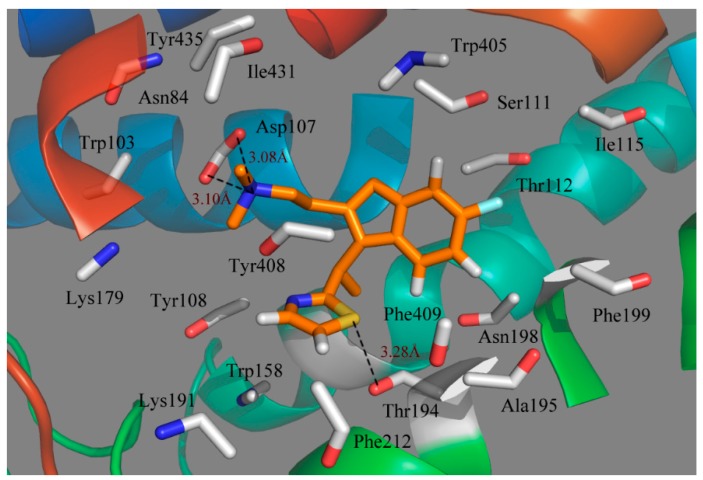
Plot of the MD-simulated structures of the binding site with compound 49. H-bonds are shown as dotted black lines; amino acid residues in the active site are represented as sticks.

Obviously, it is seen from the MD simulations with docked compound 49 in the histamine H_1_-receptor that the binding pocket interacting with the ligand is straight through the entire receptor (Figure 7). It is clearly observed that the most active compound 49 interacts with the active site of histamine H_1_ through an extensive hydrophobic region, including mainly the following residues: Tyr108, Tyr408, Trp158, Phe212, Phe409, Ala195, Phe199, Ile115, Ile431 and Trp405; while a mixture of hydrophilic amino acids Thr194, Thr112, Ser111 and Asn84 form the other side; thus, hydrophobic groups would benefit from the potency. In addition, Trp158 and Tyr435 are also located at a favorable region to form a lipophilic cavity and contribute to the binding between the antagonist and protein [38]. As seen from Figure 7, most of the key amino acids in the range of a 4.5 Å distance from the ligand on the basis of the average MD simulated complex structure are very similar to those obtained from the docking results.

Due to the molecular dynamics in the binding process, some atoms of the ligand and the protein move closer to each other, revealing the hydrogen bindings during the MD simulation course. As seen from Figure 7, the strictly conserved residue Asp107 forms an ionic interaction, *i.e.*, the so-called salt bridge, with the cationic nitrogen on the side chain of the ligand in both the docking and MD simulations, which contributed much to the ligand–receptor binding. Compound 49 also exhibits a strong binding affinity through H-bond interaction with the residue of Thr194, stabilizing the ligand at the binding pocket. Due to the rotations of the NCH_3_CH_3_ group of the ligand, the π-stacking interaction with Tyr108 is broken after MD simulations. However, at the end of the MD simulation, the observed residues suggest that the ligand moves much closer in toward the residues in the active pocket and then extends deeper into the cavity.

Actually, it is found that the MD simulation could also remedy the deficiencies from molecular docking through investigating some differences between the molecular docking and the MD simulation results.

## 3. Methodology

### 3.1. Data Source for Computational Modeling

Presently, a large dataset of 129 H_1_-antihistamines with their experimental *K_i_* values is used [26,27,28,29,30,31,32,33] for developing 3D-QSAR models. The biological activities (*K_i_* values) of these compounds range from 4.605 to 0.0003 μM. In order to be used as a dependent variable in *in silico* analyses, all of the experimental *K_i_* values were converted into the corresponding p*K_i_* (−log *K_i_*). With the aim of constructing 3D-QSAR models, the total dataset was split into two parts, including training and test sets, with a ratio of about 1/4. As a result, 96 molecules of the dataset were selected as the training set to generate the QSAR model, and the remaining 33 compounds were chosen as the test set for validating the model. Selection of the whole dataset obeys the rule that the training set should be representative of the structural diversity of the original dataset and cover approximately all ranges of p*K_i_* values. All molecules with their biological activity are listed in the Supporting Information (Table S1, Supplementary Material). Table 2 shows the representative structures of H_1_-antihistamines and their *K_i_* values.

**Table 2:** Representative structures of H_1_-antihistamines and their *K_i_* values.

No.	Structure	*K_i_* (μM)	No.	Structure	*K_i_* (μM)
001	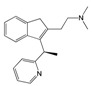	0.0004	068	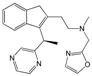	0.162
009 ^$^	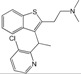	0.008	072 ^$^	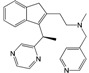	0.0049
015	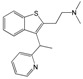	0.004	080	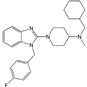	0.0044
027 ^$^	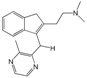	0.063	085	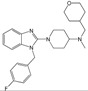	0.0039
037	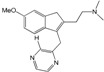	0.089	093	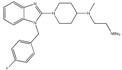	0.0029
046	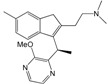	0.0027	102 ^$^	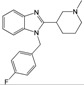	0.009
052	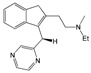	0.0072	107	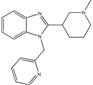	0.12
063	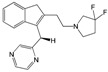	0.249	123	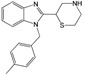	0.063

^$^ Molecules belonged to the test set.

### 3.2. Geometry Optimization and Alignment

To construct a rational 3D-QSAR model, full geometry optimizations are performed on the structures of compounds studied here. Employing the Gasteiger–Hückel method, partial atomic charges were computed and then assigned to each atom. Meanwhile, each molecule was minimized by the Powell method and the Tripos standard force [44] with a distance-dependent dielectric function. When the convergence criterion value of 0.001 kcal·mol^−1^·Å^−1^ was reached or the 1000-step minimization cycle limit was exceeded, the energy minimization was terminated. Through an iterative procedure, a partial equalization of orbital electronegativity was obtained, and the diverse charge distributions of organic molecules have gone back to the few fundamental data [45]. Therefore, for the molecules consisting of C, H, O and N atoms, their charge distributions need only 27 starting values for computation [45]. We also add this description in the manuscript.

**Figure 8:**
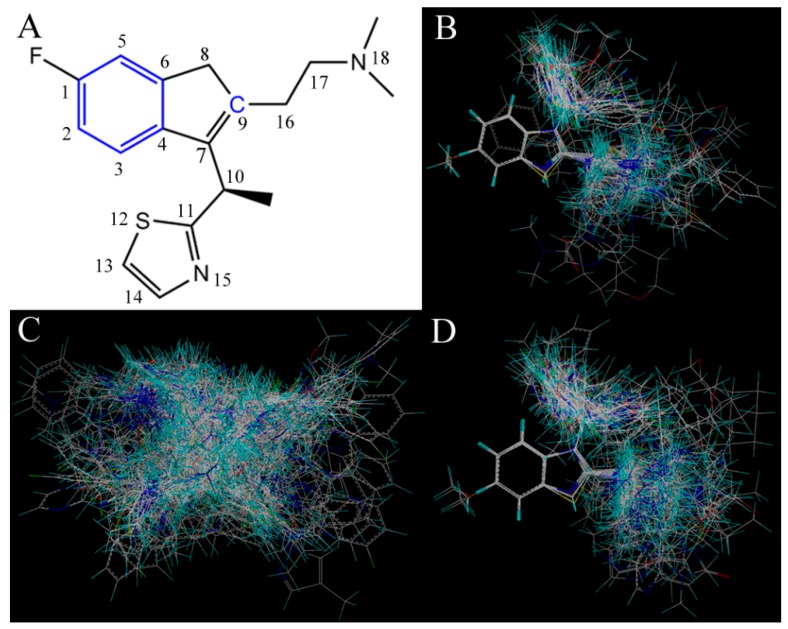
(**A**) Compound 49 used as the template molecule for alignments, with the common framework marked in blue bold. The substituent containing a protonated –NMe_2_ group at the position-18 is depicted in a red oval, which would be more desirable for potent antagonism activity; (**B**–**D**) show the results of Alignment-I, -II and -III of all molecules, respectively. All compounds in these panels are colored white for common carbon, blue for nitrogen, red for oxygen, yellow for sulfur and cyan for hydrogen atoms, respectively.

Additionally, the most important step for a QSAR model is the molecular alignment. To generate effective and reliable models, three different alignment strategies were applied in this work. Alignment-I is a ligand-based alignment scheme (Figure 8B) in which compound 49 (Figure 8A) with the highest activity was chosen as the template for alignment, and the remaining compounds were superimposed on it based on the common scaffold by using the Align Database program package embedded in SYBYL 6.9 [45]. Figure 8C presents the other approach, *i.e.*, Alignment-II. It is a receptor-based alignment, where all compounds were aligned by the optimal conformations obtained from molecular docking. With respect to Alignment-III (Figure 8D), it is a common scaffold-based alignment and similar to Alignment-I, in which all compound conformations were firstly obtained from the receptor-based alignment and then subjected to the process of Alignment-I.

### 3.3. Building Atom-Based 3D-QSAR Models

All 3D-QSAR studies were performed on Sybyl 6.9 software. CoMFA, widely-used method for constructing QSAR models, relates the biological activities of molecules with their physicochemical properties [46]. Employing an sp^3^ carbon probe atom with +1.0 charge and a 1.52 Å van der Waals radius, descriptors of steric and electrostatic fields in CoMFA were calculated on a regular lattice with a grid spacing of 2.0 Å. Both the steric and electrostatic field energies values were generated by a distance-dependent dielectric and truncated to ±30 kcal/mol [47]. CoMSIA, an extension to the CoMFA procedure, can avoid some inherent deficiencies, which arise from the fields of Lennard–Jones and Columbic potentials used in CoMFA. In CoMSIA, five physicochemical properties, *i.e.*, steric, electrostatic, hydrophobic, H-bond donor and acceptor fields, were evaluated. Using a probe atom with a radius of 2.0 Å, +1.0 charge, and hydrophobic and H-bond properties of +1, all fields were calculated at the same lattice box. A default value of 0.3 was set for the attenuation factor.

As for the differences, the Tripos force field is only utilized in CoMFA, with a distance-dependent dielectric constant in all interactions, while CoMSIA utilizes a Gaussian-type distance-dependent dielectric constant to minimize changes in atomic positions and charge potentials at the grids [48]. Additionally, the field interactions with each compounds in CoMFA were generated by the CoMFA standard scaling, while CoMSIA possesses some superiority over CoMFA, such as greater robustness in terms of shifts in region and within the alignments, as well as more intuitively interpretable contour maps [49]. Moreover, CoMSIA can avoid some inherent deficiencies generated from the steric and electrostatic fields in CoMFA. In CoMSIA, the steric and electrostatic fields near the atomic nuclei with their scale fields for PLS analyses can be rapidly changed [50].

Following descriptor calculations, partial least squares (PLS) regression analysis was used to generate effective and rational models. All of the CoMFA/CoMSIA descriptors served as independent variables, and p*K_i_* values were used as dependent variables. To evaluate the predictive power of the obtained models and to determine the optimum number of components (ONC), the cross-validation analysis was performed with the leave-one-out (LOO) method. Then, a cross-validated coefficient, *Q*^2^, was obtained, showing a glimpse of model predictive ability. Based on the highest *Q*^2^, the lowest standard error of prediction (SEP) and ONC, the final QSAR model was obtained. Subsequently, the Pearson coefficient (*R*^2^_ncv_), standard error of estimate (SEE) and *F*-test ratio were generated by the non-cross-validated analysis. Finally, based on the molecules of the test set, the predictive correlation coefficient (*R*^2^_pred_), was obtained from external validation according to the following equation:

(1) $${R_{\text{pred}}}^{2}=\frac{SD-PRESS}{SD}$$

where *SD* denotes the sum of squared deviations between the biological activities of molecules in the test set and the average activities of compounds in the training set and *PRESS* represents the sum of squared deviations between the actual and predicted activity values of molecules in the test set.

### 3.4. Analysis of Molecular Docking

To obtain the most suitable bioactive conformation and explore the interactions of ligands at the protein target’s active site, docking studies on a series of 129 compounds were carried out by using the GOLD (Genetic Optimization of Ligand Docking, Version 5.1) program package [51,52]. This docking programs is based on a powerful genetic algorithm (GA) method for obtaining bioactive conformations. Presently, the crystal structure of the histamine H_1_-receptor (PDB: 3RZE) with a resolution of 3.1 Å was downloaded from the RCSB Protein Data Bank for docking. For the protein preparation, all of the co-crystallized ligands were removed from the original protein–ligand complex at first. Then, the polar hydrogen atoms were added. Subsequently, with other parameters set at the default values, all ligands were docked into the protein’s active site, which was defined with a 10 Å radius around the ligand present in the crystal structure. Based on the criteria that the conformation gained the highest total docking score, the 10 top-scoring conformations for each ligand were retrieved and then utilized for further QSAR modeling.

### 3.5. Docking Reliability Validated by the Re-Dock Procedure

Prior to docking analysis, it is necessary to generate an acceptable reliability of the parameters specified for docking and its accuracy. Herein, a protocol was constructed by the reproduction of the X-ray complex at first. With this purpose, a known co-crystallized doxepin, which is extracted from the X-ray structure of the complex, was flexibly re-docked to the binding pocket of the histamine H_1_-receptor. As a result, the most probable binding conformation was obtained by the conformation of the molecule with the highest score. The selected re-docked binding mode was further used to search for the binding conformations to target protein for other ligands.

### 3.6. MD Simulations of Complexes

In order to validate the docking simulation and to analyze the dynamics action of the ligand, we carried out the MD simulation by using the 53a6 force field embedded in the GROMACS 4.5 software system [53]. The ligand-receptor complex was put into a cubic box and solvated with a SPC water model. The minimum distance between the protein surface and the box walls was set to 10 Å. For calculating the Coulomb and van der Waals’ interactions, the cut-off distances values were set to 1.0 and 1.4 nm, respectively. Using the PRODRG program, all parameters of the ligands specified for both the force field and the topology file were created. The charges of the ligands were computed by the Gasteiger–Hückel method [54].

Before the MD simulation, a calculation of energy minimization without any constraints using the deepest descent (SD) algorithm was performed on the entire system. Then, the system achieved equilibration at 300 K via 500 = ps MD simulations, and subsequently, a 5 = ns simulation with a time step of 2 fs was carried out. During the MD process, the simulation was performed under periodic boundary conditions with a normal pressure and temperature (NPT) ensemble at 300 K. The temperature was kept constant employing the Berendsen thermostat. The values of the isothermal compressibility were set to 4.5 × 10^−5^ bar^−1^, while the pressure was maintained at 1 bar using the Parrinello–Rahman scheme [55]. The particle mesh Ewald (PME) method [56] was used for calculating the electrostatic energy, and the linear constraint solver (LINCS) algorithm was adapted to fix all bonds involving hydrogen [57]. In addition, the cut-off distance values for computing both Coulomb and van der Waals’ interactions were 1.0 and 1.4 nm, respectively. Finally, the first 1-ns MD simulation for long equilibration was carried out, and each lasted for 5 ns to ensure the stability of the full system.

## 4. Conclusions

In the present work, using a combined computational method containing molecular docking, MD simulations and 3D-QSAR techniques, an attempt has been made to profile the structural determinants of H_1_-antihistamines for the treatment of insomnia. The combined analysis has generated a satisfactory CoMSIA model (*Q*^2^ = 0.525, *R*^2^_ncv_ = 0.891, *R*^2^_pred_ = 0.807), presenting both significant internal and external predictive capability. The contour maps generated may show a good guide for designing novel compounds with high antagonism activity. Moreover, the results derived from molecular docking and MD simulation give a detailed understanding of the binding mode of the ligands to the receptors and confirmed the key interaction features, key residues in the binding pockets.

**Figure 9:**
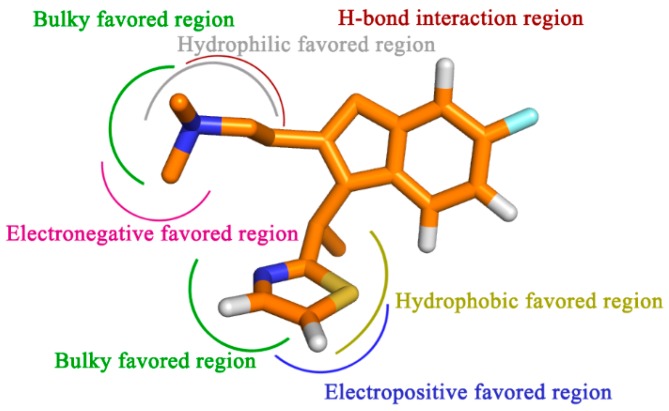
Proposed hypothetical histamine H_1_-receptor active site models. The structure-activity relationship is taken from the results of 3D-QSAR, docking and MD simulation studies for compound 49.

To sum up, by the incorporation of steric, electrostatic and hydrophobic together with HB donor groups, the information obtained from this study displays the key structural features that impact the bioactivity (Figure 9). All of the results are expected to help us to better interpret the structure-activity relationship of the H_1_-antihistamines and to give important information for aid in the design of novel potent compounds in the future.

## Supplementary Materials

Supplementary materials can be found at http://www.mdpi.com/1422-0067/17/1/129/s1.

## References

[1] Lankford A., Rogowski R., Essink B., Ludington E., Durrence H.H., Roth T. (2012). Efficacy and safety of doxepin 6 mg in a four-week outpatient trial of elderly adults with chronic primary insomnia. Sleep Med..

[2] American Academy of Family Physicians, American Psychiatric Association (1995). Diagnostic and Statistical Manual of Mental Disorders: Primary Care Version.

[3] Sullivan S.S., Guilleminault C. (2009). Emerging drugs for insomnia: New frontiers for old and novel targets. Expert Opin. Emerg. Drugs.

[4] Hart R.P., Morin C.M., Best A.M. (1995). Neuropsychological performance in elderly insomnia patients. Aging Neuropsychol. Cogn..

[5] Ohayon M.M. (2002). Epidemiology of insomnia: What we know and what we still need to learn. Sleep Med. Rev..

[6] Leung C. (2003). Neuropsychopharmacology: The fifth generation of progress. Hong Kong J. Psychiatry.

[7] Videla S., Lahjou M., Guibord P., Xu Z., Tolrà C., Encina G., Sicard E., Sans A. (2012). Food effects on the pharmacokinetics of doxylamine hydrogen succinate 25 mg film-coated tablets: A single-dose, randomized, two-period crossover study in healthy volunteers. Drugs R&D.

[8] Bovet D. (1950). Introduction to antihistamine agents and antergan derivatives. Ann. N. Y. Acad. Sci..

[9] Schutte-Rodin S., Broch L., Buysse D., Dorsey C., Sateia M. (2008). Clinical guideline for the evaluation and management of chronic insomnia in adults. J. Clin. Sleep Med..

[10] Roth T., Rogowski R., Hull S., Schwartz H., Koshorek G., Corser B., Seiden D., Lankford A. (2007). Efficacy and safety of doxepin 1 mg, 3 mg, and 6 mg in adults with primary insomnia. Sleep.

[11] Church M., Maurer M., Simons F., Bindslev-Jensen C., van Cauwenberge P., Bousquet J., Holgate S., Zuberbier T. (2010). Risk of first-generation H_1_-antihistamines: A GA2LEN position paper. Allergy.

[12] Monti J.M., Monti D. (2000). Histamine H_1_ receptor antagonists in the treatment of insomnia. CNS Drugs.

[13] Chihara Y., Sato A., Ohtani M., Fujimoto C., Hayashi T., Nishijima H., Yagi M., Iwasaki S. (2013). The effect of a first-generation H_1_-antihistamine on postural control: A preliminary study in healthy volunteers. Exp. Brain Res..

[14] Simon F.E.R., Simons K.J. (2008). H_1_ antihistamines: Current status and future directions. World Allergy Organ. J..

[15] Lin Y., Wang Y., Sima L.F., Wang D.H., Cao X.H., Chen L.G., Chen B. (2013). Design, synthesis and biological activity evaluation of desloratadine analogues as H_1_ receptor antagonists. Bioorg. Med. Chem..

[16] Suessbrich H., Waldegger S., Lang F., Busch A. (1996). Blockade of HERG channels expressed in Xenopus oocytes by the histamine receptor antagonists terfenadine and astemizole. FEBS Lett..

[17] Redfern W., Carlsson L., Davis A., Lynch W., MacKenzie I., Palethorpe S., Siegl P., Strang I., Sullivan A., Wallis R. (2003). Relationships between preclinical cardiac electrophysiology, clinical QT interval prolongation and torsade de pointes for a broad range of drugs: Evidence for a provisional safety margin in drug development. Cardiovasc. Res..

[18] Lewis D.R., Kholodovych V., Tomasini M.D., Abdelhamid D., Petersen L.K., Welsh W.J., Uhrich K.E., Moghe P.V. (2013). *In silico* design of anti-atherogenic biomaterials. Biomaterials.

[19] Wang J., Li Y., Yang Y., Zhang J., Du J., Zhang S., Yang L. (2015). Profiling the interaction mechanism of indole-based derivatives targeting the HIV-1 gp120 receptor. RSC Adv..

[20] Li P., Chen J., Wang J., Zhou W., Wang X., Li B., Tao W., Wang W., Wang Y., Yang L. (2014). Systems pharmacology strategies for drug discovery and combination with applications to cardiovascular diseases. J. Ethnopharmacol..

[21] Ru J., Li P., Wang J., Zhou W., Li B., Huang C., Li P., Guo Z., Tao W., Yang Y. (2014). TCMSP: A database of systems pharmacology for drug discovery from herbal medicines. J. Cheminform..

[22] Yu H., Chen J., Xu X., Li Y., Zhao H., Fang Y., Li X., Zhou W., Wang W., Wang Y. (2012). A systematic prediction of multiple drug-target interactions from chemical, genomic, and pharmacological data. PLoS ONE.

[23] Zhou W., Huang C., Li Y., Duan J., Wang Y., Yang L. (2013). A systematic identification of multiple toxin-target interactions based on chemical, genomic and toxicological data. Toxicology.

[24] Wang J., Li Y., Yang Y., Du J., Zhang S., Yang L. (2015). *In silico* research to assist the investigation of carboxamide derivatives as potent TRPV1 antagonists. Mol. Biosyst..

[25] Wang J., Li Y., Yang Y., Zhang S., Yang L. (2013). Profiling the Structural Determinants of Heteroarylnitrile Scaffold-Based Derivatives as Falcipain-2 Inhibitors by *In Silico* Methods. Curr. Med. Chem..

[26] Moree W.J., Li B.F., Jovic F., Coon T., Yu J., Gross R.S., Tucci F., Marinkovic D., Zamani-Kord S., Malany S. (2009). Characterization of novel selective H_1_-antihistamines for clinical evaluation in the treatment of insomnia. J. Med. Chem..

[27] Moree W.J., Jovic F., Coon T., Yu J., Li B.F., Tucci F.C., Marinkovic D., Gross R.S., Malany S., Bradbury M.J. (2010). Novel benzothiophene H_1_-antihistamines for the treatment of insomnia. Bioorg. Med. Chem. Lett..

[28] Li B.F., Moree W.J., Yu J., Coon T., Zamani-Kord S., Malany S., Jalali K., Wen J., Wang H., Yang C. (2010). Selectivity profiling of novel indene H_1_-antihistamines for the treatment of insomnia. Bioorg. Med. Chem. Lett..

[29] Huang C., Moree W.J., Zamani-Kord S., Li B.F., Tucci F.C., Malany S., Wen J., Wang H., Hoare S.R., Yang C. (2011). Influence of p*K*_a_ on the biotransformation of indene H_1_-antihistamines by CYP2D6. Bioorg. Med. Chem. Lett..

[30] Coon T., Moree W.J., Li B., Yu J., Zamani-Kord S., Malany S., Santos M.A., Hernandez L.M., Petroski R.E., Sun A. (2009). Brain-penetrating 2-aminobenzimidazole H_1_-antihistamines for the treatment of insomnia. Bioorg. Med. Chem. Lett..

[31] Lavrador-Erb K., Ravula S.B., Yu J., Zamani-Kord S., Moree W.J., Petroski R.E., Wen J., Malany S., Hoare S.R., Madan A. (2010). The discovery and structure-activity relationships of 2-(piperidin-3-yl)-1H-benzimidazoles as selective, CNS penetrating H_1_-antihistamines for insomnia. Bioorg. Med. Chem. Lett..

[32] Moree W.J., Li B.F., Zamani-Kord S., Yu J., Coon T., Huang C., Marinkovic D., Tucci F.C., Malany S., Bradbury M.J. (2010). Identification of a novel selective H_1_-antihistamine with optimized pharmacokinetic properties for clinical evaluation in the treatment of insomnia. Bioorg. Med. Chem. Lett..

[33] Ravula S.B., Yu J., Tran J.A., Arellano M., Tucci F.C., Moree W.J., Li B.F., Petroski R.E., Wen J., Malany S. (2012). Lead optimization of 2-(piperidin-3-yl)-1H-benzimidazoles: Identification of 2-morpholin-and 2-thiomorpholin-2-yl-1H-benzimidazoles as selective and CNS penetrating H_1_-antihistamines for insomnia. Bioorg. Med. Chem. Lett..

[34] Muñoz C., Adasme F., Alzate-Morales J.H., Vergara-Jaque A., Kniess T., Caballero J. (2012). Study of differences in the VEGFR2 inhibitory activities between semaxanib and SU5205 using 3D-QSAR, docking, and molecular dynamics simulations. J. Mol. Graph. Model..

[35] AbdulHameed M.D.M., Hamza A., Liu J., Zhan C.G. (2008). Combined 3D-QSAR modeling and molecular docking study on indolinone derivatives as inhibitors of 3-phosphoinositide-dependent protein kinase-1. J. Chem. Inf. Model..

[36] Shimamura T., Shiroishi M., Weyand S., Tsujimoto H., Winter G., Katritch V., Abagyan R., Cherezov V., Liu W., Han G.W. (2011). Structure of the human histamine H_1_ receptor complex with doxepin. Nature.

[37] Thangapandian S., Krishnamoorthy N., John S., Sakkiah S., Lazar P., Lee Y.N., Lee K.W. (2010). Pharmacophore modeling, virtual screening and molecular docking studies for identification of new inverse agonists of human histamine H_1_ receptor. Bull. Korean Chem. Soc..

[38] Kiss R., Kovári Z., Keserű G.M. (2004). Homology modelling and binding site mapping of the human histamine H_1_ receptor. Eur. J. Med. Chem..

[39] Wang J., Li F., Li Y., Yang Y., Zhang S., Yang L. (2013). Structural features of falcipain-3 inhibitors: An *in silico* study. Mol. Biosyst..

[40] Ohta K., Hayashi H., Mizuguchi H., Kagamiyama H., Fujimoto K., Fukui H. (1994). Site-directed mutagenesis of the histamine H_1_ receptor: Roles of aspartic acid 107, asparagine 198 and threonine 194. Biochem. Biophys. Res. Commun..

[41] Nonaka H., Otaki S., Ohshima E., Kono M., Kase H., Ohta K., Fukui H., Ichimura M. (1998). Unique binding pocket for KW-4679 in the histamine H_1_ receptor. Eur. J. Pharmacol..

[42] Bruysters M., Pertz H.H., Teunissen A., Bakker R.A., Gillard M., Chatelain P., Schunack W., Timmerman H., Leurs R. (2004). Mutational analysis of the histamine H_1_-receptor binding pocket of histaprodifens. Eur. J. Pharmacol..

[43] Wang X., Yang Q., Li M., Yin D., You Q. (2010). *In silico* binding characteristics between human histamine H_1_ receptor and antagonists. J. Mol. Model..

[44] Clark M., Cramer R.D., van Opdenbosch N. (1989). Validation of the general purpose Tripos 5.2 force field. J. Comput. Chem..

[45] Gasteiger J., Marsili M. (1980). Iterative partial equalization of orbital electronegativity—A rapid access to atomic charges. Tetrahedron.

[46] Böhm M., Stürzebecher J., Klebe G. (1999). Three-dimensional quantitative structure-activity relationship analyses using comparative molecular field analysis and comparative molecular similarity indices analysis to elucidate selectivity differences of inhibitors binding to trypsin, thrombin, and factor Xa. J. Med. Chem..

[47] Kubinyi H. (1997). QSAR and 3D QSAR in drug design Part 1: Methodology. Drug Discov. Today.

[48] Pirhadi S., Ghasemi J.B. (2010). 3D-QSAR analysis of human immunodeficiency virus entry-1 inhibitors by CoMFA and CoMSIA. Eur. J. Med. Chem..

[49] Kakarla P., Devireddy A.R., Inupakutika M.A., Cheeti U.R., Floyd J.T., Mun M.M., Vigil R.N., Hunter R.P., Varela M.F. (2014). Molecular modelling, 3D-QSAR, and drug docking studies on the role of natural anticoagulant compounds in antithrombotic therapy. Int. J. Pharm. Sci. Res..

[50] Klebe G., Abraham U., Mietzner T. (1994). Molecular similarity indices in a comparative analysis (CoMSIA) of drug molecules to correlate and predict their biological activity. J. Med. Chem..

[51] Jones G., Willett P., Glen R.C., Leach A.R., Taylor R. (1997). Development and validation of a genetic algorithm for flexible docking. J. Mol. Biol..

[52] Jones G., Willett P., Glen R.C. (1995). Molecular recognition of receptor sites using a genetic algorithm with a description of desolvation. J. Mol. Biol..

[53] Van Der Spoel D., Lindahl E., Hess B., Groenhof G., Mark A.E., Berendsen H.J. (2005). GROMACS: Fast, flexible, and free. J. Comput. Chem..

[54] SchuÈttelkopf A.W., van Aalten D.M. (2004). PRODRG: A tool for high-throughput crystallography of protein-ligand complexes. Acta Crystallogr. D Biol. Crystallogr..

[55] Parrinello M., Rahman A. (1981). Polymorphic transitions in single crystals: A new molecular dynamics method. J. Appl. Phys..

[56] Lin J.H., Perryman A.L., Schames J.R., McCammon J.A. (2002). Computational drug design accommodating receptor flexibility: The relaxed complex scheme. J. Am. Chem. Soc..

[57] Darden T., York D., Pedersen L. (1993). Particle mesh Ewald: An N·log(N) method for Ewald sums in large systems. J. Chem. Phys..

