# Supplementary material for: Computational Analysis of Structure-Based Interactions for Novel H1-Antihistamines
Source: Int J Mol Sci. 2016 Jan 19;17(1):129. doi: 10.3390/ijms17010129 (PMC4730370; doi:10.3390/ijms17010129)
Supplement: Supplementary file 1 [file ijms-17-00129-s001.pdf]

Supplementary Materials: Computational Analysis of Structure-Based Interactions for Novel H1-Antihistamines

Yinfeng Yang, Yan Li, Yanqiu Pan, Jinghui Wang, Feng Lin, Chao Wang, Shuwei Zhang and Ling Yang

Table S1. Structure of compounds with different skeletons in the data set.

No.	Structure	pK_i (μM)	No.	Structure	pK_i (μM)
1	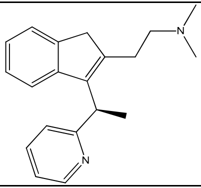	9.398	9 §	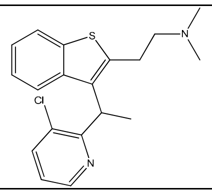	8.097
2 §	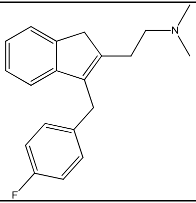	9.222	10 §	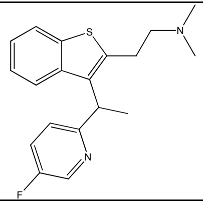	7.886
3	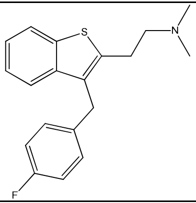	7.959	11	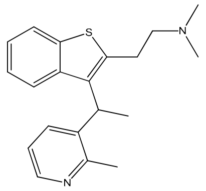	7.770
4	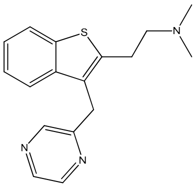	7.125	12	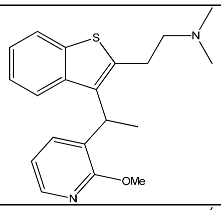	8.538
5	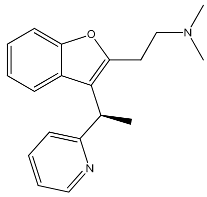	9.048	12	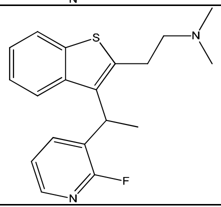	8.770
6 §	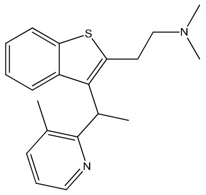	8.032	14	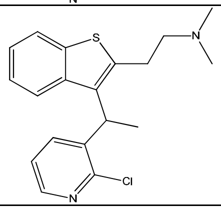	8.538
7	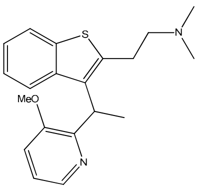	8.301	15 §	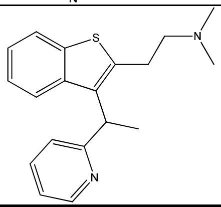	8.398

Table S1. *Cont.*

No.	Structure	pK_i (μM)	No.	Structure	pK_i (μM)
8	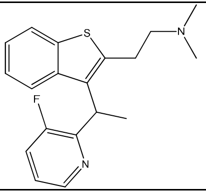	8.569	16	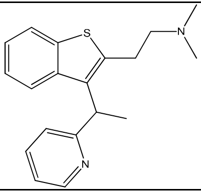	6.592
17 §	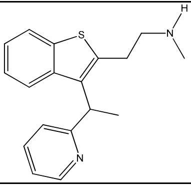	7.495	25	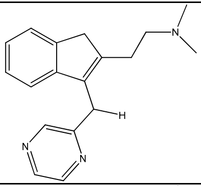	8.398
18	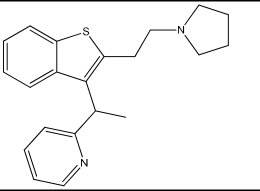	8.215	26	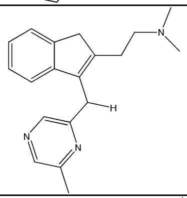	7.921
19 §	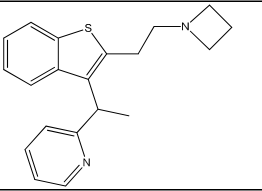	8.409	27 §	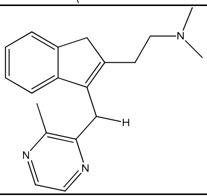	7.201
20	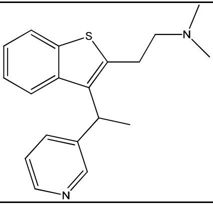	8.456	28 §	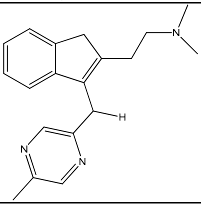	7.456
21	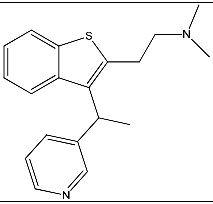	7.102	29	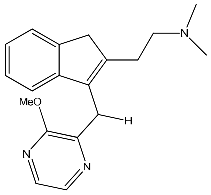	8.658
22 §	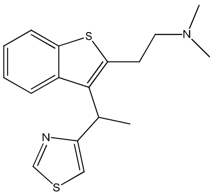	8.337	30	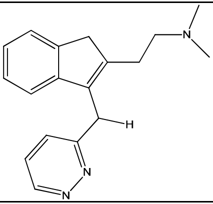	8.114
23	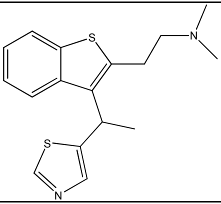	8.284	31 §	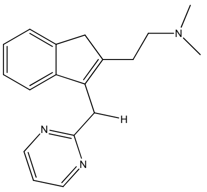	7.824
24 §	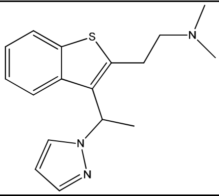	8.357	32	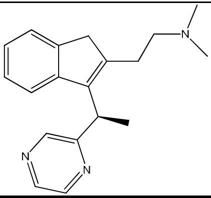	8.886

Table S1. *Cont.*

No.	Structure	pK_i (μM)	No.	Structure	pK_i (μM)
33 §	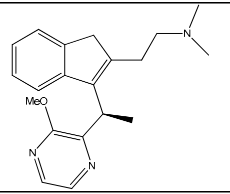	9.000	41	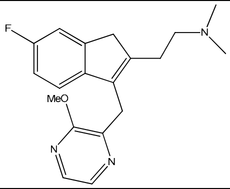	9.097
34	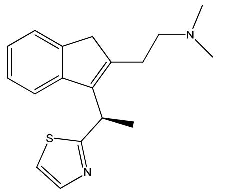	9.097	42 §	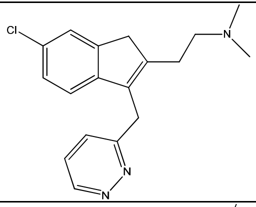	8.678
35	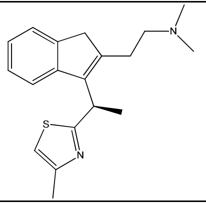	8.509	43	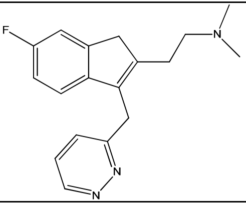	8.770
36	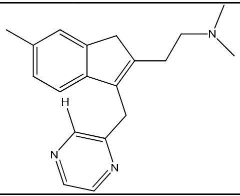	7.924	44	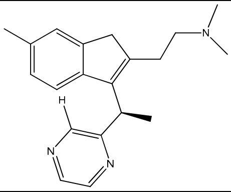	8.377
37	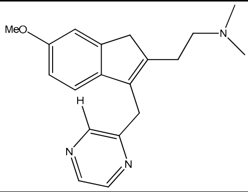	8.051	45 §	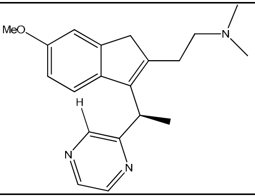	8.509
38	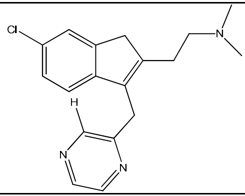	8.854	46	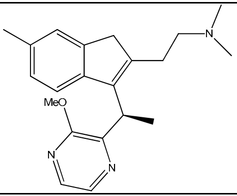	8.569
39 §	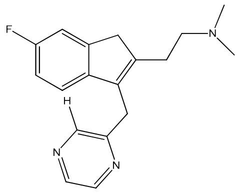	8.854	47	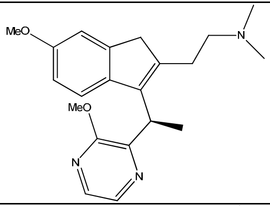	8.886
40	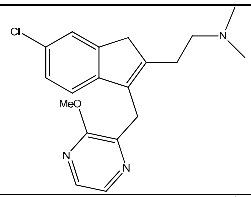	8.824	48 §	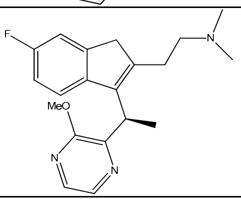	9.155
49	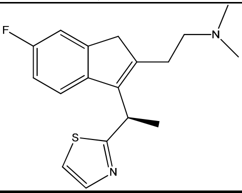	9.523	57 §	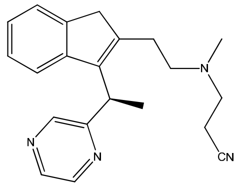	6.963

Table S1. *Cont.*

No.	Structure	pK_i (μM)	No.	Structure	pK_i (μM)
50	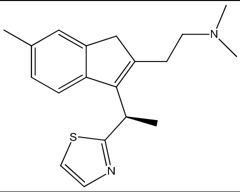	8.658	58	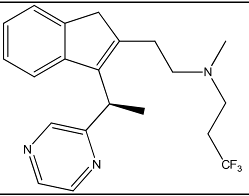	7.041
51	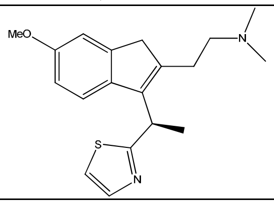	8.602	59	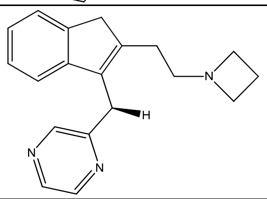	8.367
52	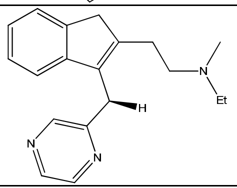	8.143	60	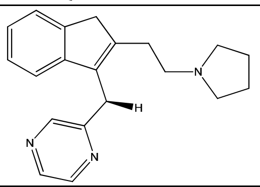	8.559
53	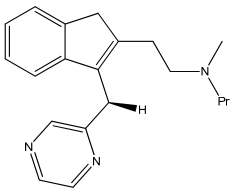	7.387	61	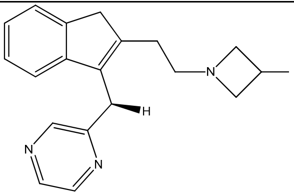	7.310
54	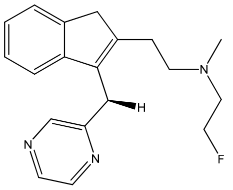	7.619	62	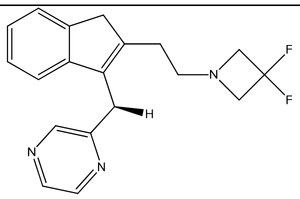	5.337
55	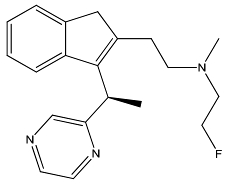	8.886	63	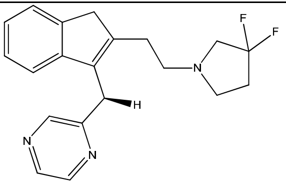	6.604
56	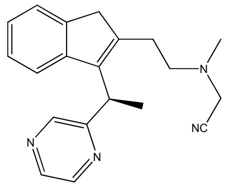	7.495	64	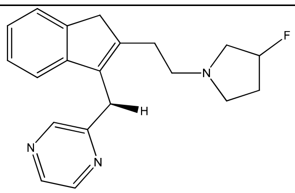	7.979
65	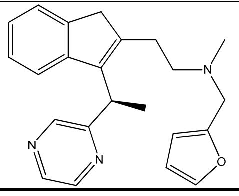	8.092	73	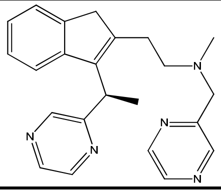	7.607

Table S1. *Cont.*

No.	Structure	pK_i (μM)	No.	Structure	pK_i (μM)
66 §	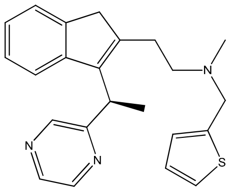	8.174	74	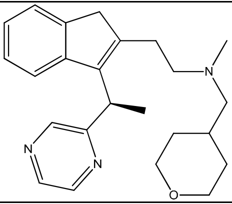	7.819
67	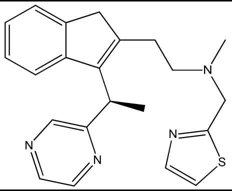	7.319	75	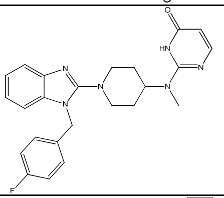	8.569
68	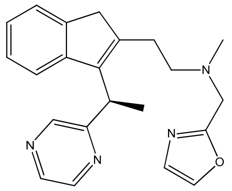	6.790	76	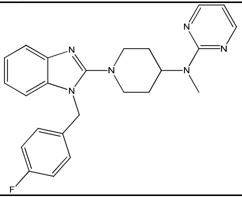	7.124
69 §	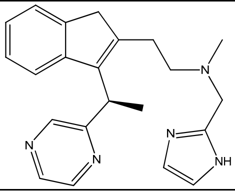	8.056	77	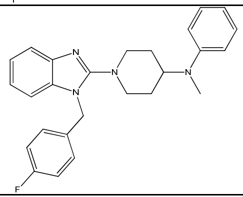	7.094
70	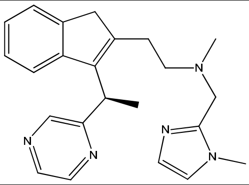	7.553	78	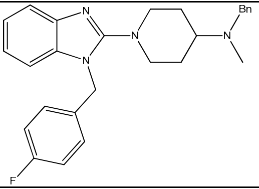	8.444
71	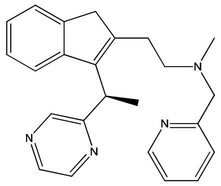	7.857	79	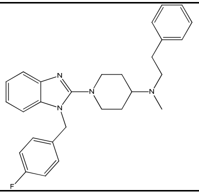	8.620
72 §	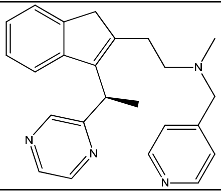	8.310	80	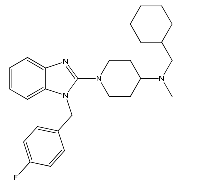	8.357
81 §	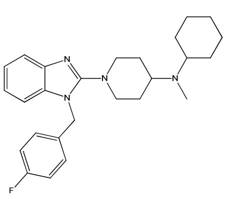	8.553	89	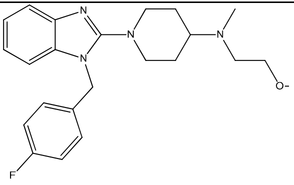	8.658
82	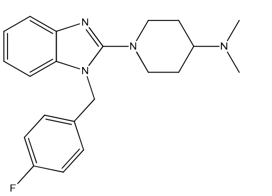	8.745	90 §	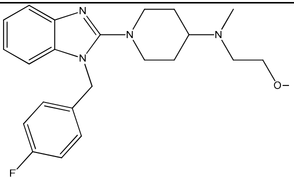	8.495

Table S1. *Cont.*

No.	Structure	pK_i (μM)	No.	Structure	pK_i (μM)
83	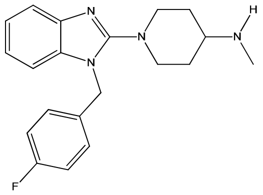	8.620	91	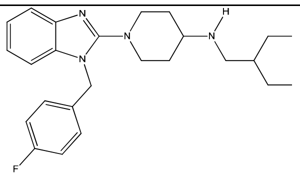	8.347
84 §	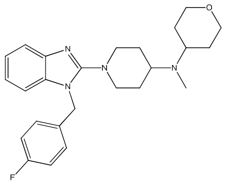	8.481	92	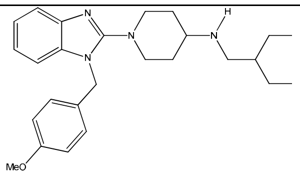	7.851
85	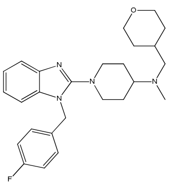	8.409	93 §	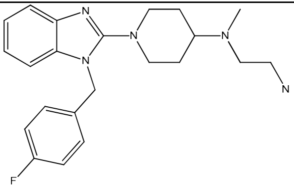	8.538
86	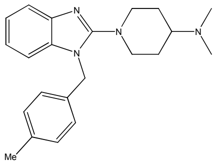	8.824	94	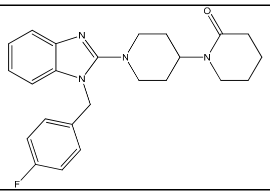	8.180
87	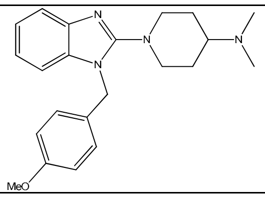	8.620	95	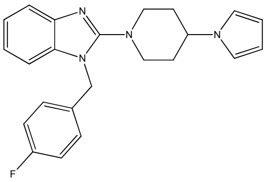	7.227
88	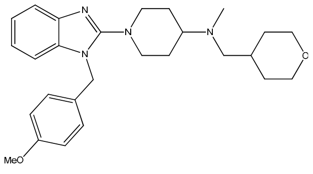	7.851	96 §	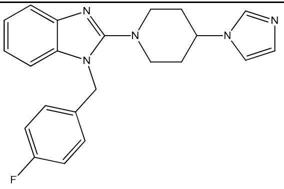	8.886
97	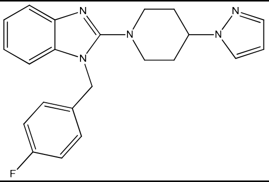	7.738	105 §	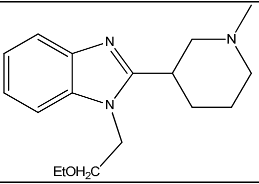	7.483
98	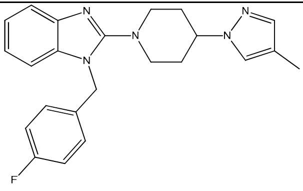	8.161	106	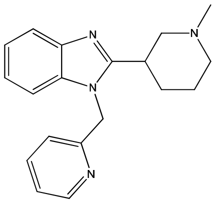	8.046

Table S1. *Cont.*

No.	Structure	pK_i (μM)	No.	Structure	pK_i (μM)
99	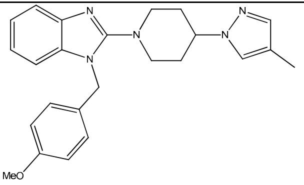	8.149	107	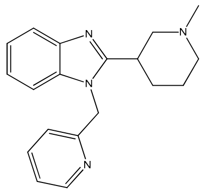	7.921
100	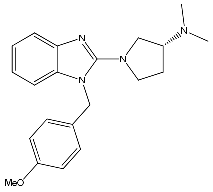	8.076	108	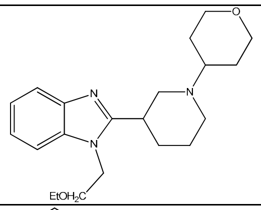	8.194
101	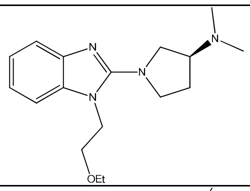	8.319	109	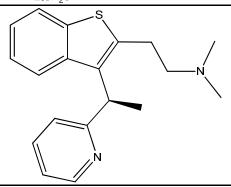	8.398
102 §	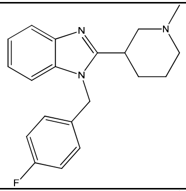	9.046	110	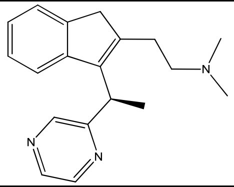	8.824
103	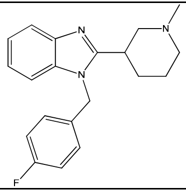	8.959	111 §	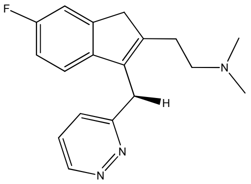	8.770
104	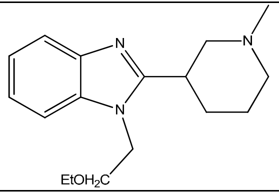	8.585	112	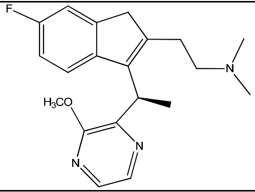	9.155
113	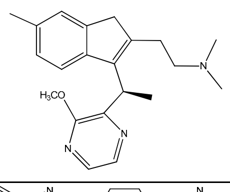	8.569	121	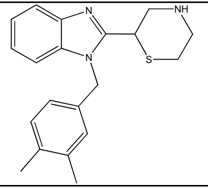	6.438
114 §	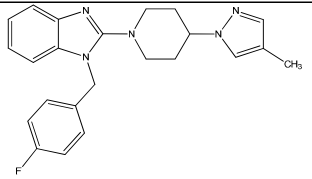	8.161	122	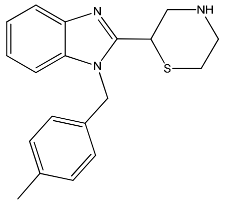	8.886
115	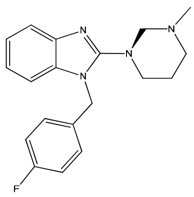	9.046	123	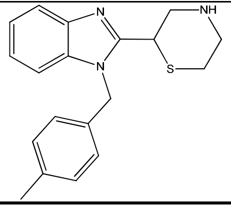	7.201

Table S1. *Cont.*

No.	Structure	pK_i (μM)	No.	Structure	pK_i (μM)
116	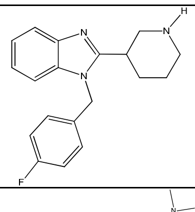	8.921	124	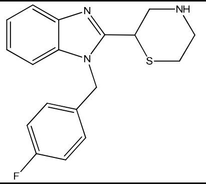	8.886
117 §	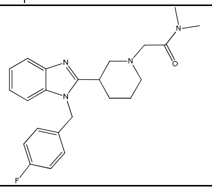	8.244	125	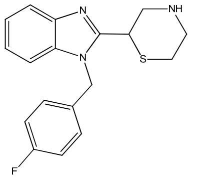	7.022
118	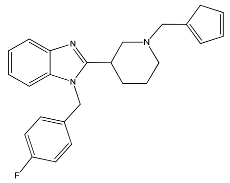	7.883	126	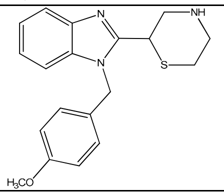	8.854
119	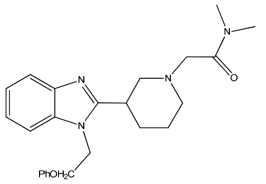	8.310	127	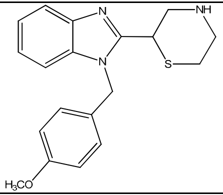	7.036
120 §	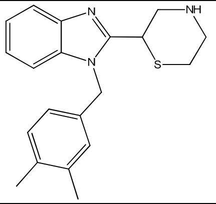	8.721	128	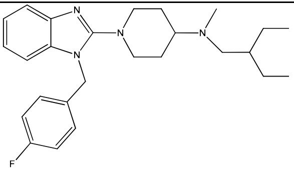	8.409
129 §	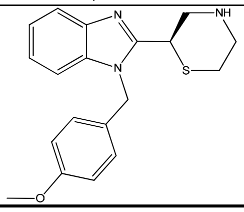	8.854			

§ Test set.
